# Midwives' Perception Towards Male Partners' Involvement in Labour Companionship: A Qualitative Study

**DOI:** 10.1111/jan.70029

**Published:** 2025-06-30

**Authors:** Tulian Chen, Yajing Wang, Zexuan Xu, Ting Wang, Tingting Fan, Guorong Jiang

**Affiliations:** ^1^ Department of Otolaryngology Shenzhen Longgang Otolaryngology Hospital & Shenzhen Otolaryngology Research Institute Shenzhen China; ^2^ The University of Edinburgh Edingburgh United Kingdom of Great Britain and Northern Ireland; ^3^ Shenzhen Hospital Affiliated, Guangzhou University of Traditional Chinese Medicine Shenzhen China; ^4^ Shenzhen Longgang Central Hospital Shenzhen China; ^5^ The Third People's Hospital of Shenzhen Shenzhen China

**Keywords:** labour companionship, male partner, midwife, qualitative study

## Abstract

**Background:**

Labour companionship is a recommendation by WHO that health authorities enable women to choose a companion during labour to ensure a safe and dignified labour experience for the birthing woman. However, most healthcare facilities in low‐ and middle‐income countries do not necessarily consider this maternal need, which hampers a positive maternal experience during labour.

**Objective:**

This study aims to examine midwives' perception towards the involvement of male partners in labour companionship.

**Methods:**

An exploratory phenomenological approach was chosen and semi‐structured interviews were used for this study.

**Results:**

The four main themes identified in this study include ‘Understanding of male partners' involvement in labour companionship’, ‘Involvement of midwives in decision‐making’, ‘Barriers to male partners' involvement in labour companionship’ and ‘Facilitators of male partners' involvement in labour companionship’.

**Conclusion and Implications:**

This study found a lack of understanding among midwives of the significance of male partners' involvement in labour companionship; and the identification of hierarchical and authoritarian leadership as a barrier to midwives' participation in decision‐making highlights the need for transformational leadership styles to empower midwives. Overall, the findings of this study can inform maternity care policy as well as resource development, education and professional training in the field of midwifery.

**Summary**

- What is already known
  ○ There are many benefits to the male partner's involvement in labour companionship.
  ○ The involvement of male partners in labour companionship does not receive enough attention.
- What this paper adds
  ○ Midwives are unaware of the role of male partners in labour companionship.
  ○ In cultures characterised by hierarchies, the role of the male partners' involvement in labour companionship is underestimated.
  ○ The practice of male partner participation in labour companionship depends on the right perception, support from the health care system, policy and education.

## 1 Introduction

Labour companionship is a recommendation by the World Health Organization (WHO) that health authorities enable women to choose a companion during labour to ensure a safe and dignified labour experience for the birthing woman (WHO 2018). According to the WHO, every woman has the right to select the person who accompanies her during childbirth, and companions include spouses, family and friends (WHO 2020). It has been suggested in the literature that the presence of a close person is very important to the birthing woman (Aune et al. 2015; Bruggemann et al. 2007; Kainz et al. 2010) and that a companion who provides physical, psychological, and emotional support during labour can help the birthing woman have a better experience (Kainz et al. 2010; Hasman et al. 2014; Bohren et al. 2019). In particular, male partners' companionship can reduce pain and loneliness, increase trust and security, and create emotional and physical well‐being (Dodou et al. 2014); it can also help increase strength and self‐confidence in coping with labour (Kainz et al. 2010) and enhance a woman's sense of control over her labour (Gibbins and Thomson 2001). In Western countries, it is usual for women to be accompanied by a male partner during labour (L. King 2017; Bryder 2015; Hasman et al. 2014). However, most healthcare facilities in low‐ and middle‐income countries do not necessarily consider and respect this maternal need (WHO 2019, 2020), or even this need is denied (Mukamurigo et al. 2017; Bohren et al. 2015), which hampers positive maternal experience during labour. For example, studies from Guatemala (Carter 2002; Carter and Speizer 2005), Myanmar (Ampt et al. 2015; Wai et al. 2015), India (Singh and Ram 2009), China (He et al. 2015), Nigeria (Oboro et al. 2011) and Brazil (Diniz et al. 2014) collectively demonstrate that male partners' companionship ranges from 16% to 87% and that this statistic also includes the part of men who accompany women to the facilities but do not remain to support women. This reflects that the involvement of male partners in labour companionship is not valued in low‐ and middle‐income countries (LMIC).

In clinical practice, midwives are the group with the greatest access to male partners to be involved in labour companionship, and midwives' perception are related to the availability of male partners to be involved in the labour process of the woman and how good or bad the experience of being involved in the process is (Krulis et al. 2021; Maluka and Peneza 2018; Emelonye et al. 2017; Hildingsson et al. 2011). It has been suggested that midwives' perception and behaviours are important in giving fathers (companions) a positive experience during labour companionship (Hildingsson et al. 2011); if midwives have positive attitudes towards male partners' involvement in labour companionship, then midwives will be positively supportive of male partners accompanying women, and midwifery support has been identified as a major need (Evans et al. 2023; Schmitt et al. 2022; Hildingsson et al. 2011). However, it has also been noted that partners who are partnered as women often receive little attention and support and are not always involved in the labour process (Maluka and Peneza 2018; Steen et al. 2012; Dolan and Coe 2011), as public health facilities in LMIC can restrict the presence of the male partner, regardless of the woman's thoughts (Bohren et al. 2019; Maluka and Peneza 2018; Emelonye et al. 2017; Mukamurigo et al. 2017; Kaye et al. 2014; Abushaikha and Massah 2013), and during pregnancy and labour, if the partner feels not being attended to and supported by the healthcare system (or midwife), he will not be able to adequately support the woman (Steen et al. 2012). Moreover, Maputle (2018) reported that no midwives encouraged the presence of a companion during labour, which could be due to staff's negative perception towards the presence of outsiders in the delivery room. This is not conducive to midwives providing quality midwifery care to women and is not conducive to promoting the further development of male partners' involvement in labour companionship in LMIC. Thus, there is a need to have a comprehensive understanding of the perception of this group (midwives) towards the involvement of male partners in labour companionship, as the perception of midwives are relevant to their provision of high‐quality midwifery care to women and to the further development of policies on male partners' involvement in labour companionship in LMIC.

There have been some studies on midwives' perception towards male partners' involvement in labour companionship (Evans et al. 2023; Khulu et al. 2022; Maputle 2018; Maluka and Peneza 2018; Emelonye et al. 2017; Rominov et al. 2017). However, most studies have concentrated on the experiences and outcomes of women or companions, with few studies singling out the perspectives of midwives and those studies that have focused primarily on Western or African contexts, with a dearth of studies that provide insight into the perception of midwives towards the involvement of male partners in labour companionship through the lens of midwives in healthcare facilities in Asian countries. This gap highlights the need for research exploring male partners' involvement in labour companionship in different cultural and organisational contexts, such as China, a cultural context imbued with Confucianism (hierarchical and patriarchal supremacy) (Ziliotti 2022). In a culture of patriarchal supremacy, men believe that the birth is a woman's business; female childbirth is considered bloody and unclean, the female puerperium is stigmatised, and men are discouraged from entering the maternity ward during their wife's labour (Littlejohn 2017; Lee 2005). In a culture of hierarchical supremacy, where obedience is emphasised, independent thinking is discouraged, and employees at the bottom of organisational hierarchies are not allowed to challenge superiors and regulations (Ziliotti 2022). Therefore, by examining midwives' perception towards the involvement of male partners in labour companionship in such contexts, it is possible to provide useful insights into how different cultural and organisational settings can influence perception and behaviours (perception as long as it includes exploring midwives' awareness, perceptions, attitudes, etc.), thereby helping to formulate implementation strategies and providing valuable insights into the development of policies for the involvement of male partners in labour companionship in Asian countries.

## 2 Methodology

### 2.1 Research Design

An exploratory phenomenological approach was chosen for this study, as it provides an in‐depth understanding and reveals the thoughts behind participants' performance behaviours (Conger 1998; Bryman 2004), and allows for an in‐depth exploration of the challenges faced by midwives when coping with the involvement of a male partner in a labour companionship, highlighting their practical experience in this particular situation. Semi‐structured interviews were used to explore and understand how midwives perceive the involvement of a male partner in labour companionship in a clinical setting. A thematic analysis was outlined by Braun and Clarke (2006) to explore participants' understanding of male partners' involvement in labour companionship, perception towards the development of male partners' involvement in labour companionship in clinical practice, and factors that inhibit or facilitate the effective development of male partners' involvement in labour companionship in clinical practice. This study followed the Comprehensive Criteria for Reporting Qualitative Research (COREQ) checklist (Tong et al. 2007).

### 2.2 Setting and Participants

This study was conducted in the meeting rooms of the obstetrics departments of two tertiary hospitals in Shenzhen, Guangdong Province. Purposive sampling was used to select participants, with inclusion criteria including midwives who had already qualified as midwives and exclusion criteria being individual posttrained midwives who had not qualified as midwives. Ten midwife participants were interviewed for this study until data saturation (Malterud et al. 2016; Kleib et al. 2024).

### 2.3 Interviews and Data Collection

The interview guide was developed by the researcher after discussion with professionals in the field. After providing participant consent, the researcher conducted a pilot interview with two participants to test the interview questions and interview duration and to ensure that the interviewer felt comfortable and knowledgeable while conducting a formal interview (DeJonckheere and Vaughn 2019). After completing the pilot interview, no new interview questions were added (Table 1).

**TABLE 1:** Semi‐structured interview questions.

Number	Questions
1	Are you aware of what male partners' involvement in labour companionship is? Are you aware of the role of the male partners' involvement in labour companionship?
2	Would you involve a male partner in accompanying a woman in labour? What is your perception towards the involvement of male partners in labour companionship?
3	What do you perceive as barriers and facilitators to the involvement of male partners in labour companionship?

The interviews for this study were conducted between November 2024 and February 2025. The head nurse of the obstetric unit was contacted in advance by the researcher to gain access to the interview site (Polit and Beck 2020) and to increase participants' authority and positive perception towards this study (Pope 2005). The participants were invited via email or WeChat by chief midwife. After providing participants' consent, the researcher arranged onsite interviews and Zoom, Tencent and WeChat videos to conduct online interviews, each of which lasted approximately 30 min. Participant recruitment stopped when no new relevant knowledge was gained (Tong et al. 2007). The audio recordings of the interviews were transcribed by a professional provider and the transcripts were returned to all participants for checking and correction; finally, the data were stored in a de‐identified format.

### 2.4 Data Analysis

**FIGURE 1:**
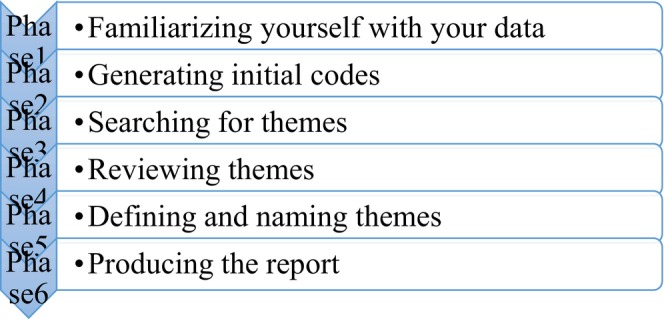
Braun and Clarke's thematic data analysis method (Braun and Clarke 2006, 87).

Braun and Clarke's (2006) thematic analysis approach was used to organise, code and sort the data and NVivo 12 software for data management. Thematic analysis ‘provides a flexible and useful research tool, which can potentially provide a rich and detailed, yet complex, account of data (Braun and Clarke 2006, 78)’; it is able to generate comprehensive and detailed data representations that are customised for the needs of qualitative research (Braun and Clarke 2006; Nowell et al. 2017; N. King 2004) (see Figure 1 for the steps involved). To gain greater understanding and familiarity with the data, two authors (Chen and Wang) reread and independently read the entire transcript. The main ideas of the participants initially emerged after both authors had developed the initial code. Differences and similarities in the approaches were then obtained after discussion among the coders (initial coding); the two authors resolved the differences (divergences) by discussing the coders with a third author (Xu). To ensure that the final code represented the data, it was reviewed by the authors; to ensure consistency between the authors when analysing the transcripts, all the meanings contained in the codes were obtained in writing. To ensure that the subthemes were distinct and coherent, the authors examined them for differences and similarities; finally, the subthemes were categorised into themes to address the research objectives (Braun and Clarke 2006).

### 2.5 Rigour

The Lincoln and Guba's (1985) principles of trustworthiness (credibility, transferability, dependability and confirmability) were considered by the authors to ensure the rigour of this study. All recordings are fully recorded and transcribed, guaranteeing credibility for the analysis. The research process provides a description of the background, process, and results of the study, guaranteeing the principle of transferability. In order to adhere to the principles of reliability and consistency, the mentor (Ph.D.) is involved throughout the research and analysis process to ensure that the results are based on the participant's perception. The first author's background of many years as a senior midwife(female) in a maternity department and her postgraduate qualifications greatly facilitated the author's ability to build a stable and trusting relationship with participants and to increase her acumen in capturing important information from the interviews, but a conscious effort was required to exclude any personal bias. The first author therefore maintained a reflective diary throughout the study to document the interview process, potential implications for data collection and analysis.

### 2.6 Ethical Considerations

Ethical waiver approval for this study was obtained from Longgang Central Hospital and Shenzhen Hospital of Guangzhou University of Traditional Chinese Medicine. The authors obtained written consent from each participant, and all data were anonymised (Mondada 2013).

## 3 Results

### 3.1 Characteristics of Participants

Ten participants (midwives were referred to as M) were interviewed for this study, and sociodemographic characteristics were also collected to help understand the background and characteristics of the participants (Table 2).

**TABLE 2:** Participant characteristics.

Participant	Age	Gender	Title	Education	Religion
M1	40	Female	Midwife manager	Ph.D.	Buddhism
M2	33	Female	Midwife manager	M.D.	Buddhism
M3	45	Female	Senior midwife	M.D.	Daoism
M4	29	Female	Senior midwife	B.D.	None
M5	28	Female	Senior midwife	B.D.	Daoism
M6	38	Female	Senior midwife	B.D.	None
M7	39	Female	Senior midwife	B.D.	None
M8	40	Female	Primary midwife	B.D.	None
M9	41	Female	Primary midwife	B.D.	Daoism
M10	42	Female	Primary midwife	B.D.	Daoism

*Note:* Daoism (Chinese system of beliefs).

Abbreviations: B.D., Bachelor's degree; M.D., Master's degree; Ph.D., Ph.D. degree.

### 3.2 Themes

Four main themes emerged from the study (‘Understanding of male partners' involvement in labour companionship’, ‘Involvement of midwives in decision‐making’, ‘Barriers to male partners' involvement in labour companionship’ and ‘Facilitators of male partners' involvement in labour companionship’) and several sub‐themes, which are collated in Table 3.

**TABLE 3:** Themes and sub‐themes.

Theme	Category	Subcategory
Understanding of male partners' involvement in labour companionship	Lack of awareness	Insufficient awareness of role
		Insufficient awareness of concepts
Involvement of midwives in decision‐making	Decision‐making is restricted	Restrictions of the hierarchical structure
		The right to involvement
Barriers to male partners' involvement in labour companionship	Bias in the context of a culture	Cultura patriarcal
		Cultural organizacional
Facilitators of male partners' involvement in labour companionship	Correct perception and support	Correct perception towards the role
		Policy and educational support

#### 3.2.1 Theme 1 Understanding of Male Partners' Involvement in Labour Companionship

The first theme related to participants' understanding of the involvement of male partners in labour companionship. Most participants had heard of male partners' involvement in labour companionship, which is prevalent in healthcare systems in Western countries, but they were unaware of the significance of the practice, that is, the role of male partners' involvement in labour companionship. Some participants articulated concepts that they thought might be relevant to the involvement of male partners in labour companionship. Therefore, this theme has two subthemes as follows:

##### 3.2.1.1 Subtheme Lack of Awareness

###### 3.2.1.1.1 Insufficient Awareness of Role

The participants had heard of the phenomenon of male partners' involvement in labour companionship, but they were unaware of the role of this practice, which illustrates the significant challenges faced by participants in recognising the positive aspects of male partners' involvement in labour companionship. Most of the participants believed that the practice of male partners' involvement in labour companionship is an outcome of the developed healthcare system in Western countries and believed that Western countries paid more attention to the humanistic needs of women after the development of healthcare resources; the participants believed that the distribution of healthcare resources in our country was uneven and that the conditions for the development of the practice of male partners' involvement in labour companionship did not yet exist. This view demonstrated a superficial understanding that male partners' involvement in labour companionship is only considered a sign of developed countries; this understanding demonstrated a lack of deeper and critical engagement with male partners' involvement in labour companionship.

I have heard about it. However, I do not understand what it does. In addition, the conditions in our hospital ensure that safe labour is most important. (Participant 2)

I know that in developed Western countries, partners are encouraged to accompany the woman. In my opinion, it works under the healthcare system in Western countries, but with the level of healthcare development and allocation of healthcare resources in our country, we should focus on healthcare safety, which is more useful than engaging in that (male partner's involvement in accompanying labour) falsehood. (Participant 7)

Further discussion revealed a consistent perception among participants that the healthcare resource situation in the country has difficulty supporting the development of male partners' involvement in labour companionship. This cognitive limitation became more apparent when participants talked about the facilitators and barriers to the development of male partners' involvement in labour companionship in the country. Instead of talking about factors that are intrinsically linked to the benefits of male partners' involvement in labour companionship, participants often associated it with setting challenges such as understaffing, maintaining a sterile environment in the delivery room, and long shifts.

The delivery room is a sterile environment, and with one person in charge of the labor, I truly do not have the spare energy to teach a person how to be a companion. Issues outside the scope of work can affect midwives and their performance. (Participant 1)

Often, labour is something that requires a midwife to focus for long periods of time, and working long hours can sometimes affect concentration, yet they have to divide half of their energy between caring for the woman's companion … This was one of the key barriers to midwives wanting to develop chaperoning. (Participant 7)

These findings illustrated the participants' narrow interpretation of male partners' involvement in labour companionship, which focused on the midwives' perceptions of themselves as completely dominating the delivery room rather than working in partnership with the male partner.

###### 3.2.1.1.2 Insufficient Awareness of Concepts

While there was a lack of awareness of male partners' involvement in labour companionship, participants were able to demonstrate an intuitive understanding of key aspects, including aspects of decision‐making, support for the midwife and the relationship with the male partner. These findings suggest that participants may have been unconscious but were able to recognise the importance of male partners' accompaniment in reducing birth accidents and enhancing maternal satisfaction.

Some participants linked the involvement of male partners in labour companionship to effective decision‐making to enhance midwife‐family cooperation, noting that it plays a key role in the timely informed consent and decision‐making of families in the event of exceptional discordance. This reflected the understanding that the presence of a male partner involves active participation in the decision‐making process and enhances cooperation with the midwife.

Is it a type of Doula delivery? Is it just that the companion becomes the husband of the woman? If so, it can help the midwives and increase the safety of the labour process. (Participant 5)

In unexpected situations, it is something that can effectively work with the midwife to make decisions together so that it can be done in a safe and quick way. (Participant 1)

A participant emphasised the relational aspect of the male partner's involvement in labour companionship, highlighting its importance in working with the midwife. This relationship sees the involvement of the male partner in labour companionship as a supportive partnership, which is important to the midwifery team, and midwives can enhance the collaborative process by encouraging and supporting the involvement of the male partner.

I believe that the role of the male partner in accompanying the birth can become a partnership for the midwife, which focuses more on the role of the companion than the woman or the midwife. (Participant 3)

#### 3.2.2 Theme 2 Involvement of Midwives in Decision‐Making

When participants were asked about the involvement in decision‐making situations where male partners were allowed to be present, they did so mainly by stating their perception towards explaining the relevant hospital or departmental regulations to male partners. This was because, midwives' involvement in decision‐making was limited by power and hierarchy differentials, but they did know the right of male partners to be involved in labour companionship. The subtheme is described below:

##### 3.2.2.1 Subtheme Decision‐Making Is Restricted

###### 3.2.2.1.1 Restrictions of the Hierarchical Structure

The bureaucracy (hierarchical structure) that characterises China's healthcare system greatly restricted midwives' participation in decision‐making about involving male partners in labour companionship, which indicated the failure of the midwife community to be empowered and the failure of higher levels of power to value the further development of male involvement in labour companionship. This restriction kept midwives working strictly within the boundaries of their regulations and cannot do anything contrary to hospital rules unless it is necessary. This perspective illustrated the challenges midwives face in navigating organisational hierarchies outside of established rules, which can lead to midwives being held accountable within the hierarchical structure and further limits the possibilities for male partners to participate in the development of labour companionship.

First, the delivery room is a place full of sterile objects, and there is a need for asepsis. Furthermore, there are no hospital rules for allowing family members to be present at birth, and I have no authority to allow their partners to come into the delivery room. Unless I make a report to the head nurse and the leader agrees, I cannot break the hospital's rules without the head nurse's consent, or I will be held accountable. (Participant 4)

The direct imposition of authority also illustrated the top‐down way to decision‐making in the participants' healthcare system, in which orders from superiors should not be refused or challenged. This reflected a culture of decision‐making where it is also possible to restrict both groups (the companion group and the midwife group), with superiors expecting orders to be carried out without enter, reflecting the bureaucratic nature of leadership in a number of cases (authoritarian nature).

What the leaders mean is that the higher authority has not given the relevant instructions to allow the husband of the woman to enter the delivery room to accompany the woman, and even if he asks for it, you cannot agree to let him in. It is the higher authority; we just have to do what the higher authority says, do not ask so many questions. (Participant 5)

In addition, some participants noted the dominance of obstetricians over midwives in making decisions about the involvement of male partners in labour companionship, illustrating the traditional role in the healthcare hierarchy, where the doctor usually has the final say over the power of the midwife group, both in terms of clinical decision‐making for woman and in terms of humanistic care. One participant's response revealed the marginalisation of the midwifery community in decision‐making regarding the involvement of male partners in labour companionship, exemplifying the structural attribution of power within healthcare.

The decision to participate in a paternity with a male partner does not depend entirely on the midwife when I say so. Rather, it depends more on the doctor, whose word is often more effective. (Participant 6)

These insights demonstrated an organisational culture that marginalises midwives in the decision‐making process, particularly in relation to decisions about the midwifery care of the woman and access to some of their family's rights. The development of the involvement of male partners in labour companionship is systematically challenged by rigid hierarchical structures and entrenched traditional roles, achieved by limiting the participation of the midwifery community in decision‐making.

###### 3.2.2.1.2 The Right to Involvement

The participants emphasised a number of roles for male partners in labour companionship, rooted in their first‐hand knowledge of women's needs. The participants emphasised the usefulness of working with a partner to improve medical safety and the quality of midwifery care.

I think the male partner is very important when working with the midwife to make decisions during labour because the male partner is someone very close to the woman, and he is just as important as the midwife or the doula…. The midwife must take the initiative to get the partner to cooperate with her … Finally, it is important to make the experience good for both the birthing person and the companion. (Participant 5)

To illustrate the role of male partner involvement in labour companionship, one midwife described how a midwife in difficult labour instructed her partner to cooperate with the midwife, and the final outcome was a smooth birth.

I remember being in a woman's labour who asked for a caesarean section as soon as she started to feel the pain of contractions because she did not want to endure the pain of labour for a long time, but the woman did not have an indication for surgery (caesarean section). We then suggested that the male partner be allowed into the delivery room to comfort and dissuade her. The doctor agreed, and half an hour later, the woman agreed to attempt her own labour, which ended in an uneventful delivery. (Participant 6)

The midwives also advocated for the right of male partners to be present throughout their partner's labour, especially if they felt that this method might be helpful to the woman. (Participant 7)

In fact, in most cases, the presence of a male partner was beneficial to the woman; after all, there are women who always want to have someone around to accompany and support them when going through such a major life event. (Participant 1)

Although with the insights, midwives faced hierarchical resistance that weakens the midwives' participation in making decisions to involve a male partner in labour companionship.

#### 3.2.3 Theme 3 Barriers to Male Partners' Involvement in Labour Companionship

This theme explored the barriers to male partners' involvement in labour companionship and encompassed a variety of barriers that indirectly or directly affect male partners' involvement in labour companionship, for example, culturally and in the setting. Although the participants mentioned a variety of factors, the authors selectively included those most relevant to male partners' involvement in labour companionship on the basis of the authors' analyses. Notably, some of the barriers, such as maintaining a sterile environment in the delivery room, understanding and long shifts, were discussed under the theme of ‘Understanding of male partners' involvement in labour companionship’. The direct relevance of these findings to male partners' involvement in labour companionship was low (due to the limited conceptual understanding of the participants) and was therefore not emphasised.

##### 3.2.3.1 Subtheme Bias in the Context of a Culture

###### 3.2.3.1.1 Cultura Patriarcal

In China's patriarchal culture, childbirth is often considered a woman's business, and very few husbands actively request that they be involved in accompanying their wives during labour. In addition, in this patriarchal culture, the delivery room is considered bloody, and contact with it can lead to misfortune. In this cultural bias, midwives advocating for male partners to participate in labour companionship are challenged by cultural factors.

…At the entrance of the labour room were the husband and mother‐in‐law, and I asked the husband to go inside the labour room and take her to the ward. Her mother‐in‐law immediately stopped me, saying that it was unlucky for a man to go into the delivery room. (Participant 10)

Several times, I could not find the family members to sign, and finally determined that the husband was a businessman and could not touch anything related to the delivery room (a Chinese culture that believes that the blood in the delivery room would attract bad luck), so he sent her in and left. (Participant 1)

###### 3.2.3.1.2 Cultural Organizacional

The participants' words reveal a general underestimation of the role of male partners' involvement in labour companionship in professional settings. This trend is indicative of a wider organisational culture that elevates the healthcare profession while marginalising the role of male partners' involvement in labour companionship. Discussions of the role of healthcare professionals predominantly emphasised the roles of doctors and midwives at the expense of the importance of the male partner. This neglect was summarised by the sentiment that the role of male partners' involvement in labour companionship was few valued or discussed.

No one was talking about male partners' companionship; in fact, it was enough for doctors and midwives to ensure safe labour. (Participant 7)

This environment led to challenges in the recognition and development of male partners' involvement in labour companionship, despite their important role in increasing the safety of labour and improving the quality of midwifery care.

In fact, the presence of a doctor and midwife is the only thing that matters during labour; male partner's attendance is not important and is just an icing on the cake like doula. (Participant 5)

As mentioned above, delivery rooms in a patriarchal context are unpopular, and the development of male partners' involvement in labour is challenged by the cultural context. This subtheme revealed that this cultural context also clearly influences the values of the healthcare system and health organisations towards the involvement of male partners in labour companionship. Analyses of participant feedback revealed systemic barriers to male partner support from health facilities, particularly the lack of professionals and relevant courses to guide male partners to become competent companions. For example, many hospitals have maternity classes but no paternity classes. This lack largely contributes to the underestimation of the role and necessity of male partners' involvement in labour companionship within the healthcare system.

In fact, there are no such materials or specialised personnel focused on training male partners to participate in labour companionship. (Participant 2)

Training courses are mostly for pregnant women, and there are no courses for male partners. (Participant 5)

It can only be said that husbands are exposed to relevant information when accompanying their wives in maternity courses. (Participant 8)

#### 3.2.4 Facilitators of Male Partners' Involvement in Labour Companionship

In this theme, the participants discussed the factors they felt were important in facilitating male partners' involvement in labour companionship. Considering facilitators, they recognised that some healthcare system cultures need to be changed and that new perceptions and policies are to be expected and introduced.

##### 3.2.4.1 Subtheme Correct Perception and Support

###### 3.2.4.1.1 Correct Perception Towards the Role

The correct perception of the role of male partner involvement in labour companionship was an important facilitator. The participants emphasised that communicating some correct knowledge about male partners' involvement in labour companionship to midwives or those with relevant exposure to labour and delivery in the healthcare system would significantly change their perception, that is, increase their positive perception towards male partners' involvement in labour companionship. As noted by participants, correct knowledge can directly influence the acceptability of male partners' involvement in labour companionship. Widespread acceptance and advocacy by healthcare professionals associated with childbirth can contribute more positively to the perception and acceptance of male partners' involvement in labour companionship, as healthcare professionals are professionals and representatives.

I think there are a number of factors, including whether we (midwives) get it right (male partner chaperoning); if we get it right, then we naturally accept and support it and communicate the benefits to the pregnant woman and her family. (Participant 4)

The participants' lack of awareness of male partners' involvement in labour companionship was also highlighted, as this lack of awareness meant that there was no way to talk about it more and support it. Overall, the analyses revealed a consensus among the participants that a correct understanding of male partners' involvement in labour companionship is an important factor in its development and implementation.

If we can all recognise that it (male partner's involvement in labour) is good for both mothers and midwives, we are willing to support its clinical spread. Otherwise, we will not take the risk. (Participant 9)

This subtheme also emphasised the need for healthcare providers to provide professional training for male partners as a group as an important facilitator of male partner involvement in labour companionship. The participants highlighted the need for the healthcare system to help male partners move away from the idea that birth is a woman's business or the idea that a man entering the labour room will bring misfortune to himself and that providing them with professional training in labour companionship would be a key facilitator; participants felt that awareness‐raising and acceptance of training by male partners would lead to greater acceptance and participation of male partners in labour companionship.

In fact, we only play a leading role. The husband's correct understanding and acceptance of his participation in labour companionship is the most important, as his role cannot be replaced. When the partner of the woman is able to understand it correctly and has been trained in the knowledge related to labour companionship (acquiring the relevant skills), they are the ones who are best able to promote labour companionship in clinical practice. (Participant 6)

Husbands need to be made to feel that labour is not a woman's business alone. Childbirth is a great event, and husbands should be active in accompanying them rather than avoiding them if they feel they will incur misfortune. (Participant 8)

The importance of training for male partners also needs to be recognised in healthcare settings, and effective training programmes can help to align the goals of midwives and companions.

Therefore, I think it would be good for both partners and midwives if hospitals could provide training for male partners in this area. (Participant 2)

###### 3.2.4.1.2 Policy and Educational Support

According to the participants, policy and educational support were factors that directly contributed to the involvement of male partners in labour companionship. The participants emphasised that the top‐down clinical introduction of provisions to allow/encourage male partners to volunteer to be present at birth and appropriate incentives for partners to be present during labour would directly promote male partners' involvement in labour companionship in practice.

The direct introduction of hospitals to allow husbands to be present throughout the labour force could have an immediate impact. (Participant 10)

…Relevant incentives for husbands who have completed their time with their wives in labour. (Participant 5)

## 4 Discussion

This study explored male partners' involvement in labour companionship from a midwife's perspective, concentrate on ‘Understanding of male partners' involvement in labour companionship’, ‘Involvement of midwives in decision‐making’, ‘Barriers to male partners' involvement in labour companionship’ and ‘Facilitators of male partners' involvement in labour companionship’ in the clinical setting.

The participants' lack of understanding of male partners' involvement in labour companionship was expected, which is consistent with studies that noted a lack of involvement of Asian male partners in their wives' maternal health care and in accompanying their wives during labour and that both male partners' involvement in maternal health care, and labour companionship have been described as neglected and inadequate researched topics (Haque Nohri et al. 2022; Nyang'au et al. 2021). The perception of participants regarding male partners' involvement in labour companionship around the prioritisation of current healthcare resources and challenges in the setting. The factors underlying the failure of male partners' involvement in labour companionship to spread into clinical practice included patriarchal cultural bias, the limited involvement of midwives in decision‐making related to male involvement in labour companionship, and a lack of support from healthcare systems and organisational structures. These factors are consistent with studies that have identified a lack of support from healthcare organisations (both at the healthcare facility and policy level) (Khulu et al. 2022; Maluka and Peneza 2018; Lewis et al. 2015), a low status of midwives in the healthcare system, no important aspects of autonomy, decision‐making power and respectful cooperation (i.e., midwives are not empowered to participate in relevant decision‐making) (Dahlen et al. 2022) and the influence of traditional cultural biases (Khulu et al. 2022; Lewis et al. 2015) in the practice of male partners' involvement in labour companionship. However, these essential factors were not initially included in participants' conceptualisations of male involvement in labour companionship; for example, when midwives were asked what facilitating factors were conducive to the introduction of male partner involvement in labour companionship or barriers to the introduction of male partner involvement in labour companionship, they responded with factors that were more related to the prioritisation of healthcare resources in the healthcare system and the working setting rather than the male partner factors (e.g., maintaining a sterile environment in the delivery room, understaffing, long shifts, etc.); although these factors are important to the safety and experience of labour, they are not necessarily related to the involvement of the male partner in labour companionship (e.g., long shifts, understaffing, maintaining a sterile environment in the delivery room would not be factors related to or directly influencing the involvement of the male partner in labour companionships). We found that ‘the factors that affect men's involvement in maternal health care services may be categorised into cultural factors, socioeconomic factors, health facility factors, interspouse communication and perceptions men have on maternal health services (Lwanga et al. 2017, 7)’. In the end, the participants identified other factors associated with male partners' involvement in labour companionships, such as cultural bias, a lack of recognition of the value of male partners' involvement in labour companionships, a lack of support from the health system and organisational structures, the need for the benefits of male partners' involvement in labour companionships to be correctly perceived, and support from policy and education as significant barriers or facilitators. However, in most cases, participants overlapped the concept and benefits of doula delivery with the benefits of male partners' involvement in labour companionships. Therefore, the concept and benefits of male partners' involvement in labour companionship need to be incorporated into midwifery education and integrated into routine midwifery care to increase midwives' understanding and knowledge of the impact of male partners' involvement in labour and labour safety in practice.

The participants' decision‐making about the involvement of a male partner in labour companionship was highly influenced by hierarchies within the organisational system (e.g., power differentials between midwives and doctors or between midwives and managers). This is in line with study suggesting that in hierarchically dominated healthcare organisational cultures, where doctors have a high level of decision‐making power, midwives or nurses have limited participation in decision‐making and are severely marginalised (Zarbiv et al. 2025; Noyes 2022; Dahlen et al. 2022). Hierarchical relationships and power imbalances between midwives and doctors or midwives and midwifery managers were reflected in some participants' expressions, such as ‘do what your superiors say, do not ask so many questions?’, and ‘doctors' words are often more effective.’ It was evident from the participants' statements that the constraints on midwives' participation in decision‐making about the involvement of male partners in labour companionship led to their negative clinical practice responses. For example, ‘unless the leader or doctor agrees, the midwife does not have any authority to allow male partners to enter the delivery room to accompany woman in the absence of any hospital regulations for doing so’. The lack of support from healthcare organisations was also revealed to be a major barrier to male partners' involvement in labour companionship. Furthermore, these narratives indicated that participants' settings typically adopted authoritarian leadership practices rather than empowering leadership practices. As Robbins et al. (2021, 329) stated, ‘An autocratic style is that of a leader who typically tends to centralise authority, dictate work methods, make unilateral decisions and limit employee participation’. This finding reinforced the fact that participants' decision‐making about male partners' involvement in labour companionship is heavily influenced by the specific leadership style that exists in the organisational setting (Robbins et al. 2021) and is heavily marginalised. Therefore, encouraging change in the hierarchical system of unequal power and empowering midwives to participate in decision‐making may be an effective way to promote midwives' participation in decision‐making regarding male partners' involvement in labour companionship, consequently, the further development of male partners' involvement in labour companionship.

Another major barrier to male partner involvement in labour companionship is the lack of recognition of the role of the male partner in labour companionship compared with the lack of support from the healthcare system. As seen in the theme of the participants' comments on ‘barriers to male involvement in labour companionship’, the participants' comments on the male partner's companionship highlight a general underestimation of the role of the male partner's involvement in labour companionship in the professional context. This is because the allocation of healthcare resources within the organisational system (scarcity) determines that there is still a need to prioritise the safety of labour and delivery over the needs of the companion (Jayasundara et al. 2024); therefore, the organisational system recognises the professional status of the healthcare practitioner compared with the companion while marginalising the role of the male partner. Multiple participants' comments discussed the dominant role of doctors and midwives during labour, for example, the presence of a doctor and midwife during labour was sufficient. For some participants, the midwife was the main player in ensuring safe labour, taking on the main tasks, with the male partner mainly taking on tasks not related to the safety of the labour. This insight implied that male partners' involvement in labour companionship is considered unimportant, which may hinder the practice and development of male partners' involvement in labour companionship. This is consistent with Dahlen et al.'s (2022) study, where midwives in under resourced or flawed organisational systems prioritised safe childbirth over the best care for women and other needs (e.g., the need for a companion). Some participants also mentioned that the role and importance of the doctor and midwife in the labour process was much greater than the involvement of the male partners and that the male partner's involvement in labour companionship was just an ‘icing on the cake’. The participants seemed to fail to understand that male partners' involvement in labour companionship is important, which is in line with research on perceptions of male partners' involvement in labour companionship and suggests that the education of midwives and the development of policies to facilitate the active involvement of male partners should be considered (Khulu et al. 2022). Therefore, it is important to raise awareness among midwives and healthcare professionals about the importance of the role of male partners' involvement in labour companionship. In addition, enhancing equality between healthcare professionals (midwives and doctors) and male partners in terms of training, policy support and educational measures to allow and encourage the practice of male partner companionship can help redefine the role to achieve a state of equilibrium characterised by partnership rather than marginalising the role of the male partner's involvement in labour companionship (Lewis et al. 2015).

## 5 Limitations

There are also limitations to this study. Most Chinese healthcare facilities contain predominantly female midwives, resulting in limited gender representation in the participant group, which may have influenced thematic insights. Cultural representation and gender balance could be sought in future research. Despite these limitations, this study provides valuable insights into the development of male partners' involvement in labour companionship in the context of midwifery in China, a topic that has rarely been investigated.

## 6 Conclusion

This study provides insights into the midwifery field in China regarding the midwifery community's perception towards male partners' involvement in labour companionship, revealing opportunities and challenges within the field. The difference in perception towards male partners' involvement in labour companionship was that male partners' involvement in labour companionship is part of the promotion of the field of midwifery in China, which addresses gaps in the existing research and provides a new perspective on the development of the field of midwifery. The identification of hierarchical and authoritarian leadership as a barrier to midwives' participation in decision‐making highlights the need for transformational leadership styles to empower midwives to facilitate the development of male partners' involvement in labour companionship. Moreover, the exploration of the facilitators and barriers to male partners' involvement in labour companionship creates new avenues for enhancing midwifery education and the development of the midwifery profession, emphasising the need to integrate the concept of male partner involvement in companionship into midwifery curricula. The training of midwives to male partners' involvement in labour companionship can provide midwives with a balanced understanding of the role of male partners' involvement in labour companionship, thereby enhancing their contribution to work. Overall, the findings of this study can inform maternity care policy as well as resource development, education and professional training in the field of midwifery to promote inclusive practices in organisational cultures.

In addition, the themes we identified, although rooted in the Chinese context, cut across geo‐cultural boundaries and illustrate the universality of the experiential aspects of maternity care. For example, the underestimation of male partners' involvement in labour companionship reflected a common misconception that may hinder the development of midwifery care within the same cultural system. By addressing this misconception, this study advocates that midwifery teams with similar cultural systems should reposition male partners' involvement in labour companionship as an integral part of improving the quality of midwifery care and maternal experience to build more effective maternity services across cultures. Furthermore, limiting midwives' participation in decision‐making processes related to paternity due to leadership and hierarchical structures is a challenge for healthcare systems. The findings of this study call for healthcare leaders with similar cultural backgrounds to those in this study to take action to empower midwives by adopting a more inclusive leadership model. In this way, the important role of male partners' involvement in labour companionship is recognised, which can lead to high‐quality midwifery care and a better birth experience for women. Ultimately, this study helps reduce the dearth of literature on male partners' involvement in labour companionship in particular cultures (e.g., cultures of hierarchical and authoritarian leadership) and helps to inform further research to explore differences in male partners' involvement in labour companionship in diverse organisational and cultural contexts. This will help in the development of universally applicable strategies to further promote male partners' involvement in labour companionship in the field of obstetrics.

## Author Contributions

Tulian Chen, Zexuan Xu, Yajing Wang and Tingting Fan contributed substantially to the conception and design of the study, or the acquisition of data, or the analysis and interpretation of data. Tulian Chen, Zexuan Xu, Yajing Wang, Tingting Fan, Ting Wang and Guorong Jiang were involved in drafting the manuscript or revising it critically for important intellectual content. All authors provided final approval of the version to be published. Each author participated sufficiently in the work to take public responsibility for the content. All authors agreed to be accountable for all aspects of the work, ensuring that questions related to the accuracy or integrity of any part of the work are appropriately investigated and resolved.

## Conflicts of Interest

The authors declare no conflicts of interest.

## Data Availability

The data that support the findings of this study are available from the corresponding author upon reasonable request.

## References

[jan70029-bib-0001] Abushaikha, L. , and R. Massah . 2013. “Perceptions of Barriers to Paternal Presence and Contribution During Childbirth: An Exploratory Study From Syria.” Birth 40, no. 1: 61–66. 10.1111/birt.12030. 24635426

[jan70029-bib-0002] Ampt, F. , M. M. Mon , K. K. Than , et al. 2015. “Correlates of Male Involvement in Maternal and Newborn Health: A Cross‐Sectional Study of Men in a Peri‐Urban Region of Myanmar.” BMC Pregnancy and Childbirth 15: 1–11. 26013564 10.1186/s12884-015-0561-9 PMC4445797

[jan70029-bib-0003] Aune, I. , H. Marit Torvik , S. T. Selboe , A. K. Skogås , J. Persen , and U. Dahlberg . 2015. “Promoting a Normal Birth and a Positive Birth Experience—Norwegian Women's Perspectives.” Midwifery 31, no. 7: 721–727. 10.1016/j.midw.2015.03.016. 25907004

[jan70029-bib-0004] Bohren, M. A. , B. O. Berger , H. Munthe‐Kaas , and Ö. Tunçalp . 2019. “Perceptions and Experiences of Labour Companionship: A Qualitative Evidence Synthesis.” Cochrane Database of Systematic Reviews 3, no. 3: CD012449. 10.1002/14651858.CD012449.pub2. 30883666 PMC6422112

[jan70029-bib-0005] Bohren, M. A. , J. P. Vogel , E. C. Hunter , et al. 2015. “The Mistreatment of Women During Childbirth in Health Facilities Globally: A Mixed‐Methods Systematic Review.” PLoS Medicine 12, no. 6: e1001847. 10.1371/journal.pmed.1001847. 26126110 PMC4488322

[jan70029-bib-0006] Braun, V. , and V. Clarke . 2006. “Using Thematic Analysis in Psychology.” Qualitative Research in Psychology 3, no. 2: 77–101. 10.1191/1478088706qp063oa.

[jan70029-bib-0007] Bruggemann, O. M. , M. A. Parpinelli , M. J. Osis , J. G. Cecatti , and A. S. Neto . 2007. “Support to Woman by a Companion of Her Choice During Childbirth: A Randomized Controlled Trial.” Reproductive Health 4: 5. 10.1186/1742-4755-4-5. 17612408 PMC1936417

[jan70029-bib-0008] Bryder, L. 2015. “Fathers and Hospital Childbirth in New Zealand.” Social History of Medicine 28, no. 4: 725–741.

[jan70029-bib-0009] Bryman, A. 2004. “Qualitative Research on Leadership: A Critical but Appreciative Review.” Leadership Quarterly 15, no. 6: 729–769.

[jan70029-bib-0010] Carter, M. 2002. “Husbands and Maternal Health Matters in Rural Guatemala: Wives' Reports on Their Spouses' Involvement in Pregnancy and Birth.” Social Science & Medicine 55, no. 3: 437–450. 10.1016/s0277-9536(01)00175-7. 12144151

[jan70029-bib-0011] Carter, M. W. , and I. Speizer . 2005. “Salvadoran Fathers' Attendance at Prenatal Care, Delivery, and Postpartum Care.” Revista Panamericana de Salud Pública/Pan American Journal of Public Health 18, no. 3: 149–156. 10.1590/s1020-49892005000800001. 16269116

[jan70029-bib-0012] Conger, J. A. 1998. “Qualitative Research as the Cornerstone Methodology for Understanding Leadership.” Leadership Quarterly 9, no. 1: 107–121.

[jan70029-bib-0013] Dahlen, H. G. , D. Drandic , N. Shah , and F. Cadee . 2022. “Supporting Midwifery Is the Answer to the Wicked Problems in Maternity Care.” Lancet Global Health 10, no. 7: e951–e952. 35714641 10.1016/S2214-109X(22)00183-8

[jan70029-bib-0014] DeJonckheere, M. , and L. M. Vaughn . 2019. “Semistructured Interviewing in Primary Care Research: A Balance of Relationship and Rigour.” Family Medicine and Community Health 7, no. 2: e000057. 32148704 10.1136/fmch-2018-000057 PMC6910737

[jan70029-bib-0015] Diniz, C. S. G. , E. d'Orsi , R. M. S. M. Domingues , et al. 2014. “Implementation of the Presence of Companions During Hospital Admission for Childbirth: Data From the Birth in Brazil National Survey.” Cadernos de Saúde Pública 30: S140–S153. 10.1590/0102-311x00127013 25167174

[jan70029-bib-0016] Dodou, H. D. , D. P. Rodrigues , E. M. Guerreiro , M. V. C. Guedes , P. N. Lago , and N. S. de Mesquita . 2014. “The Contribution of the Companion to the Humanisation of Delivery and Birth: Perceptions of Puerperal Women.” Escola Anna Nery 18: 262–269. 10.5935/1414-8145.20140038.

[jan70029-bib-0017] Dolan, A. , and C. Coe . 2011. “Men, Masculine Identities and Childbirth.” Sociology of Health & Illness 33, no. 7: 1019–1034. 10.1111/j.1467-9566.2011.01349.x. 21561461

[jan70029-bib-0018] Emelonye, A. U. , T. Pitkäaho , A. Aregbesola , and K. Vehviläinen‐Julkunen . 2017. “Barriers to Spousal Contribution to Childbirth Pain Relief in Nigeria.” International Nursing Review 64, no. 4: 568–575. 10.1111/inr.12330. 27933605

[jan70029-bib-0019] Evans, K. , P. Pallotti , H. Spiby , C. Evans , and J. Eldridge . 2023. “Supporting Birth Companions for Women in Labor, the Views and Experiences of Birth Companions, Women and Midwives: A Mixed Methods Systematic Review.” Birth 50, no. 4: 689–710. 10.1111/birt.12736. 37593922

[jan70029-bib-0020] Gibbins, J. , and A. M. Thomson . 2001. “Women's Expectations and Experiences of Childbirth.” Midwifery 17, no. 4: 302–313. 10.1054/midw.2001.0263. 11749063

[jan70029-bib-0021] Haque Nohri, M. U. , P. Akhter Memon , M. Ali Mallah , K. Bux Mangiro , A. Ali Malik , and M. Ahmed Soomro . 2022. “Male Involvement in Maternity Care and Birth Preparedness of Their Spouse.” Pakistan BioMedical Journal 5, no. 7: 284–289. 10.54393/pbmj.v5i7.514.

[jan70029-bib-0022] Hasman, K. , H. Kjaergaard , and B. A. Esbensen . 2014. “Fathers' Experience of Childbirth When Nonprogressive Labour Occurs and Augmentation Is Established. A Qualitative Study.” Sexual & Reproductive Healthcare 5, no. 2: 69–73. 10.1016/j.srhc.2014.02.001. 24814441

[jan70029-bib-0023] He, H. G. , K. Vehviläinen‐Julkunen , X. F. Qian , D. Sapountzi‐Krepia , Y. Gong , and W. Wang . 2015. “Fathers' Feelings Related to Their Partners' Childbirth and Views on Their Presence During Labour and Childbirth: A Descriptive Quantitative Study.” International Journal of Nursing Practice 21, no. S2: 71–79. 10.1111/ijn.12339. 26125575

[jan70029-bib-0024] Hildingsson, I. , L. Cederlöf , and S. Widén . 2011. “Fathers' Birth Experience in Relation to Midwifery Care.” Women and Birth 24, no. 3: 129–136. 10.1016/j.wombi.2010.12.003. 21216684

[jan70029-bib-0025] Jayasundara, D. M. C. S. , I. A. Jayawardane , S. D. S. Weliange , T. D. K. M. Jayasingha , and T. M. S. S. B. Madugalle . 2024. “Impact of Continuous Labor Companion‐ Who Is the Best: A Systematic Review and Meta‐Analysis of Randomised Controlled Trials.” PLoS One 19, no. 7: e0298852. 10.1371/journal.pone.0298852. 39042637 PMC11265680

[jan70029-bib-0026] Kainz, G. , M. Eliasson , and I. von Post . 2010. “The Child's Father, an Important Person for the Mother's Well‐Being During the Childbirth: A Hermeneutic Study.” Health Care for Women International 31, no. 7: 621–635. 10.1080/07399331003725499. 20526927

[jan70029-bib-0027] Kaye, D. K. , O. Kakaire , A. Nakimuli , M. O. Osinde , S. N. Mbalinda , and N. Kakande . 2014. “Male Involvement During Pregnancy and Childbirth: Men's Perceptions, Practices and Experiences During the Care for Women Who Developed Childbirth Complications in Mulago Hospital, Uganda.” BMC Pregnancy and Childbirth 14: 1–8. 24479421 10.1186/1471-2393-14-54 PMC3916059

[jan70029-bib-0028] Khulu, Z. A. , M. L. M. Sengane , and T. Mudau . 2022. “Midwives' Perspectives Regarding Involvement of Male Partners During Pregnancy at a Maternal Health Facility in Eswatini.” Africa Journal of Nursing and Midwifery 24, no. 2: 19. 10.25159/2520-5293/12096.

[jan70029-bib-0029] King, L. 2017. “Hiding in the Pub to Cutting the Cord? Men's Presence at Childbirth in Britain c.1940s–2000s.” Social History of Medicine 30, no. 2: 389–407. 10.1093/shm/hkw057. 29713116 PMC5914339

[jan70029-bib-0030] King, N. 2004. “21—Using Templates in the Thematic Analysis of Text.” In Essential Guide to Qualitative Methods in Organisational Research, edited by C. Cassell and G. Symon , 256–270. Sage.

[jan70029-bib-0031] Kleib, M. , A. Arnaert , L. M. Nagle , R. Sugars , and D. da Costa . 2024. “Newly Qualified Canadian Nurses' Experiences With Digital Health in the Workplace: Comparative Qualitative Analysis.” JMIR Medical Education 10, no. 1: e53258. 39159452 10.2196/53258 PMC11369539

[jan70029-bib-0032] Krulis, J. , M. König‐Bachmann , and C. Zenzmaier . 2021. “Einflussfaktoren auf das väterliche Erleben der Geburt im Kreißsaal: Eine qualitative Studie [Factors Influencing the Paternal Experience of Birth in the Labour Ward: A Qualitative Study].” Zeitschrift für Geburtshilfe und Neonatologie 225, no. 2: 167–175. 10.1055/a-1204-2212. 32942323

[jan70029-bib-0034] Lee, J.‐D. 2005. “Childbirth in Early Imperial China.” Nan Nü 7: 216–286. 10.1163/156852605775248658.

[jan70029-bib-0035] Lewis, S. , A. Lee , and P. Simkhada . 2015. “The Role of Husbands in Maternal Health and Safe Childbirth in Rural Nepal: A Qualitative Study.” BMC Pregnancy and Childbirth 15: 162. 10.1186/s12884-015-0599-8. 26239123 PMC4523911

[jan70029-bib-0036] Lincoln, Y. S. , and E. G. Guba . 1985. Naturalistic Inquiry. Sage.

[jan70029-bib-0037] Littlejohn, L. J. 2017. “Confucianism: How Analects Promoted Patriarchy and Influenced the Subordination of Women in East Asia.” Young Historians Conference. 9. https://pdxscholar.library.pdx.edu/younghistorians/2017/oralpres/9/.

[jan70029-bib-0038] Lwanga, H. , L. Atuyambe , H. Sempewo , A. Lumala , and R. N. B. Byaruhanga . 2017. “An Exploratory Study of Men's Companionship, Perceptions and Experiences During Pregnancy and Delivery in Uganda.” BMC Pregnancy and Childbirth 17, no. 1: 196. 10.1186/s12884-017-1385-6. 28629332 PMC5477342

[jan70029-bib-0039] Malterud, K. , V. D. Siersma , and A. D. Guassora . 2016. “Sample Size in Qualitative Interview Studies: Guided by Information Power.” Qualitative Health Research 26, no. 13: 1753–1760. 26613970 10.1177/1049732315617444

[jan70029-bib-0040] Maluka, S. O. , and A. K. Peneza . 2018. “Perceptions on Male Involvement in Pregnancy and Childbirth in Masasi District, Tanzania: A Qualitative Study.” Reproductive Health 15: 68. 10.1186/s12978-018-0512-9. 29678184 PMC5910565

[jan70029-bib-0041] Maputle, M. S. 2018. “Support Provided by Midwives to Women During Labour in a Public Hospital, Limpopo Province, South Africa: A Participant Observation Study.” BMC Pregnancy and Childbirth 18: 210. 10.1186/s12884-018-1860-8. 29871607 PMC5989402

[jan70029-bib-0042] Mondada, L. 2013. “Ethics in Action: Anonymization as a Participant's Concern and a Participant's Practice.” Human Studies 37, no. 2: 179–209.

[jan70029-bib-0043] Mukamurigo, J. , A. Dencker , J. Ntaganira , and M. Berg . 2017. “The Meaning of a Poor Childbirth Experience—A Qualitative Phenomenological Study With Women in Rwanda.” PLoS One 12, no. 12: e0189371. 10.1371/journal.pone.0189371. 29220391 PMC5722369

[jan70029-bib-0044] Nowell, L. S. , J. M. Norris , D. E. White , and N. J. Moules . 2017. “Thematic Analysis: Striving to Meet the Trustworthiness Criteria.” International Journal of Qualitative Methods 16, no. 1: 1609406917733847.

[jan70029-bib-0045] Noyes, A. L. 2022. “Navigating the Hierarchy: Communicating Power Relationships in Collaborative Health Care Groups.” Management Communication Quarterly 36, no. 1: 62–91. 10.1177/08933189211025737.

[jan70029-bib-0046] Nyang'au, R. A. M. , M. Wanzala , and T. Were . 2021. “Male Partner Involvement in Promoting Antenatal Care and Skilled Delivery Attendance in Bumula Sub‐County, Kenya.” European Journal of Medical and Health Sciences 3, no. 5: 43–51. 10.24018/ejmed.2021.3.5.978.

[jan70029-bib-0047] Oboro, V. O. , A. O. Oyeniran , S. E. Akinola , and A. I. Isawumi . 2011. “Attitudes of Nigerian Women Toward the Presence of Their Husband or Partner as a Support Person During Labor.” International Journal of Gynecology & Obstetrics 112, no. 1: 56–58. 21056414 10.1016/j.ijgo.2010.07.033

[jan70029-bib-0048] Polit, D. , and C. Beck . 2020. Essentials of Nursing Research: Appraising Evidence for Nursing Practice. Lippincott Williams & Wilkins.

[jan70029-bib-0049] Pope, C. 2005. “Conducting Ethnography in Medical Settings.” Medical Education 39, no. 12: 1180–1187. 10.1111/j.1365-2929.2005.02330.x. 16313576

[jan70029-bib-0061] Robbins, S. P. , M. A. Coulter , D. A. DeCenzo , and M. Woods . 2021. Management: The Essentials. 5th ed. Pearson.

[jan70029-bib-0050] Rominov, H. , R. Giallo , P. D. Pilkington , and T. A. Whelan . 2017. “Midwives' Perceptions and Experiences of Engaging Fathers in Perinatal Services.” Women and Birth 30, no. 4: 308–318. 10.1016/j.wombi.2016.12.002. 28094187

[jan70029-bib-0051] Schmitt, N. , S. Striebich , G. Meyer , A. Berg , and G. M. Ayerle . 2022. “The Partner's Experiences of Childbirth in Countries With a Highly Developed Clinical Setting: A Scoping Review.” BMC Pregnancy and Childbirth 22, no. 1: 742. 10.1186/s12884-022-05014-1. 36192684 PMC9528111

[jan70029-bib-0052] Singh, A. , and F. Ram . 2009. “Men's Involvement During Pregnancy and Childbirth: Evidence From Rural Ahmadnagar, India.” Population Review 48, no. 1: 83–102. https://muse.jhu.edu/article/264738.

[jan70029-bib-0053] Steen, M. , S. Downe , N. Bamford , and L. Edozien . 2012. “Not‐Patient and Not‐Visitor: A Metasynthesis Fathers' Encounters With Pregnancy, Birth and Maternity Care.” Midwifery 28, no. 4: 362–371. 10.1016/j.midw.2011.06.009. 21820778

[jan70029-bib-0054] Tong, A. , P. Sainsbury , and J. Craig . 2007. “Consolidated Criteria for Reporting Qualitative Research (COREQ): A 32‐Item Checklist for Interviews and Focus Groups.” International Journal for Quality in Health Care 19, no. 6: 349–357. 17872937 10.1093/intqhc/mzm042

[jan70029-bib-0055] Wai, K. M. , A. Shibanuma , N. N. Oo , T. J. Fillman , Y. M. Saw , and M. Jimba . 2015. “Are Husbands Involving in Their Spouses' Utilisation of Maternal Care Services?: A Cross‐Sectional Study in Yangon, Myanmar.” PLoS One 10, no. 12: e0144135. 26641891 10.1371/journal.pone.0144135 PMC4671588

[jan70029-bib-0056] World Health Organization . 2018. “WHO Recommendations on Intrapartum Care for a Positive Childbirth Experience.” Monthly Report on Dracunculiasis Cases, January–May 2017. 30070803

[jan70029-bib-0057] World Health Organization . 2019. “Why Having a Companion During Labour and Childbirth May Be Better for You.” https://www.who.int/news/item/19‐03‐2019‐why‐having‐a‐companion‐during‐labour‐and‐childbirth‐may‐be‐better‐for‐you.

[jan70029-bib-0058] World Health Organization . 2020. “Companion of Choice During Labour and Childbirth for Improved Quality of Care.” Monthly Report on Dracunculiasis Cases, January–May 2017.

[jan70029-bib-0059] Zarbiv, G. , S. Perlman , and M. E. Ellen . 2025. “Barriers and Facilitators for Implementation of Continuity of Midwife Care: A Review of Reviews.” Women and Birth 38: 101892. 10.1016/j.wombi.2025.101892. 40037130

[jan70029-bib-0060] Ziliotti, E. 2022. “Questions for Hierarchical Confucianism.” Review of Politics 84, no. 3: 329–349. 10.1017/S0034670522000304.

